# Reviewed article: Mour EB, Catelano BA, Aguiar FP, Lima H, França PHC. Survival of critically ill people with COVID-19 and acute kidney injury undergoing hemodialysis in public and private hospitals in Joinville: a cohort study, 2020-2021. Epidemiol Serv Saude. 2025:34;20240025. doi: 10.1590/S2237-96222025v34e20240025.en

**DOI:** 10.1590/S2237-96222025v34e20240025.a

**Published:** 2025-04-07

**Authors:** Wildo Navegantes de Araújo

**Affiliations:** 1 Universidade de Brasilia, Ceilândia, DF,Brazil

## Peer review A

## Questionnaire

Does the manuscript contain new and significant information to justify publication? Yes

Does the Abstract (Summary) clearly and accurately describe the content of the article? Not applicable

Is the problem significant and concisely stated? Yes

Are the methods described comprehensively? Yes

Are the interpretations and conclusions justified by the results? Not applicable

Is adequate reference made to other work in the field? Yes

## Manuscript Structure

Length of article is: Adequate

Number of tables is: Adequate

Number of figures is: Adequate

**Please state any conflict(s) of interest that you have in relation to the review of this paper (state “none” if this is not applicable).**

None

**Interest:** Average

**Quality:** Good

**Originality:** Average

**Overall:** Good

**Recommendation:** Major Revision

## Comments

O artigo contribui com o conhecimento do tema, porém precisa incorporar principalmente os fatores relacionados a melhoria do método (anexo) e rever na discussão a exploração dos resultados do ponto de vista de validade externa, dado que foram estudados participantes de pesquisa de três unidades de saúde de uma região de saúde. A conclusão apontado principalmente no resumo precisa ser mais especificamente baseado nos resultados obtidos.

